# Development of an efficient antimicrobial susceptibility testing method with species identification by Nanopore sequencing of 16S rRNA amplicons

**DOI:** 10.1371/journal.pone.0262912

**Published:** 2022-02-03

**Authors:** Yuto Kawai, Naoya Ozawa, Takako Fukuda, Noriyuki Suzuki, Kazuki Mikata

**Affiliations:** Bioscience Research Laboratory, Sumitomo Chemical Co., Ltd, Osaka, Japan; Nitte University, INDIA

## Abstract

While amplicon sequencing of 16S rRNA is a common method for studying microbial community, it has been difficult to identify genera and species using next-generation sequencers to examine some regions (e.g., V3–V4 of 16S rRNA) because of the short read lengths. However, the advent of third-generation sequencers has made it possible to analyze the full length of the 16S rRNA gene, which allowed for species level identification at low cost. In this study, we evaluated the accuracy of the identification with a third-generation sequencer, MinION from Oxford Nanopore Technologies, using nine indigenous bacteria that can pose problems with food poisoning and opportunistic infections as an example. We demonstrated that *Enterococcus faecalis* and *Enterococcus hirae* could be identified at the species level with an accuracy of 96.4% to 97.5%. We also demonstrated that the absolute counts of various bacteria could be determined by spiking the sample with a bacterium as an internal standard. Then, we tested whether this convenient bacterial identification method could evaluate the antibiotic sensitivities of multiple bacteria simultaneously. In order to evaluate antimicrobial susceptibility, a mock community, an artificial mixture of the nine bacterial strains, was prepared and cultured in the presence of the antibiotics ofloxacin or chloramphenicol, and the 16S rRNAs were analyzed by using Nanopore sequencer. We confirmed that antibiotic-induced cell count reductions could be measured simultaneously by quantifying the abundances of various bacteria in the mock community before and after culture. It was thus shown that the antibiotic sensitivities of multiple bacteria could be evaluated simultaneously, with distinction made between bactericidal action and bacteriostatic action. This methodology would allow rapid evaluation of antibiotic activity spectrum at the species level containing a wide variety of bacteria, such as biofilm bacteria and gut microbiota.

## Introduction

Bacteria exist in complex communities in all environments, bringing about various human health issues, including food contamination [1], tooth decay [2], and diseases mediated by gut microbiota change [3]. Antimicrobial resistance is a global health and development threat to humankind. Since misuse and overuse of antimicrobials are the main drivers in the development of drug-resistant pathogens, only appropriate antimicrobials should be used. On the other hand, since some bacteria are beneficial to humans [4], it is desirable to suppress only pathogenic bacteria in human microbiome for probiotics or cosmetics. Antimicrobial susceptibility is usually evaluated by using the disc diffusion method [5,6] or the microdilution method [7,8]. These methods visually check the proliferation of bacteria cultured in the presence of antibiotics in agar medium or liquid medium, offering advantages of very low cost and no need for special equipment. However, because the antibiotic effect is evaluated for each test strain, analysis and culture of a large number of test strains is time-consuming and it is impossible to know whether the antimicrobial effect is bactericidal or bacteriostatic (simply suppresses bacterial proliferation without causing cell death). The distinction between bactericidal activity and bacteriostatic activity is important in industry for food preservatives and healthcare [9]. The time-kill methodology [10,11] is a convenient method for bactericidal activity analysis where reductions in the number of viable antibiotic-treated bacteria are determined by colony counting on agar medium. However, it takes much more time than the disc diffusion and the microdilution method. Therefore, a convenient method for concurrently evaluating the bactericidal effects of antibiotics is needed.

Recent advances in sequencing technology have made it possible to comprehensively analyze bacterial taxonomy and abundance in large diverse bacterial populations. At present, sequencing a particular region of the 16S rRNA gene using a next-generation sequencer (NGS) is the most commonly used method for microbial community analysis [12]. It has proven to be a powerful strategy for the taxonomic classification of bacteria, but too short read length makes species level identification impossible. Utilizing the single-molecule analysis principle, the third-generation sequencers have the great advantage of long read analysis lengths. The Nanopore sequencer MinION particularly provides rapid analyses at low cost. This technology has made it possible to analyze the full length of the 16S rRNA gene and has made convenient identification at the species level. Although their error rates are higher than those of the NGS [13], Nanopore sequencers have been used for rapid detection of pathogenic bacteria [14], microbial community analysis at high resolution based on its long-read capability [15,16], and analysis of soil and water bacterial community [17,18]. These studies suggest that the accuracy of Nanopore sequencing is sufficient for measurement of bacterial taxonomy and abundance.

**Fig 1:**
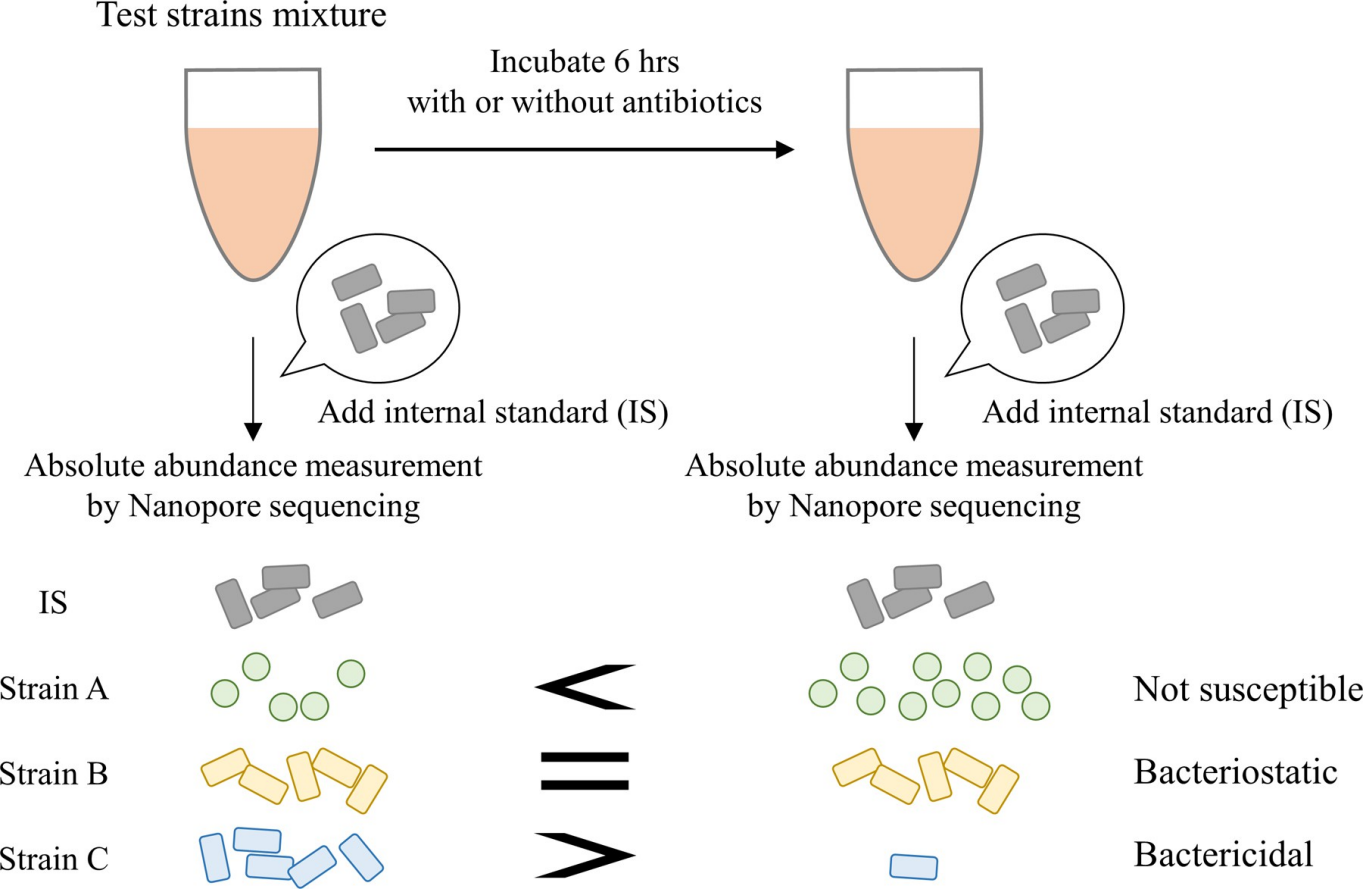
Design of antimicrobial susceptibility testing methodology. A culture containing several test strains were prepared, and the absolute abundances of each test strains before and after exposure to antimicrobials was measured by 16S rRNA amplicon sequencing. Internal standard bacteria were added to the culture before DNA extraction.

In this study, we tested whether measuring bacterial abundance by sequencing instead of colony counting could be applied to antibiotic susceptibility test (Fig 1). The absolute abundances of test strains in culture before and after exposure to antibiotics were measured by using Nanopore sequencing of 16S rRNA amplicons. Although conventional analysis of microbes by 16S rRNA amplicon sequencing only allows us to measure the relative abundance of each bacterium, this limitation was overcome by spiking samples with a known bacterium as the internal standard [19]. This analysis allowed us to classify the test strains into three groups based on antibiotic susceptibility: normally proliferating bacteria (no antibacterial activity), non-proliferating bacteria (bacteriostatic activity), and dead or dying bacteria (final count less than initial cell count; bactericidal activity).

## Materials and methods

### Strains and medium

Nine test strains and one internal standard strain were used. The following test strains were obtained from the American Type Culture Collection (ATCC); *Kocuria rhizophila* (ATCC 9341), *Escherichia coli* (ATCC 33588), *Enterococcus hirae* (ATCC 8043), *Bacillus subtilis* (ATCC 6051), *Enterococcus faecalis* (ATCC 47077), *Streptococcus mutans* (ATCC 25175), *Listeria innocua* (ATCC33090), and *Staphylococcus epidermidis* (ATCC12228). *Ralstonia solanacearum* (T-11 strain) [20] was isolated from Tomato plants. *Lactobacillus sakei* (NBRC 15893) used for internal standard was obtained from the National Institute of Technology and Evaluation (NBRC). All these strains were cultured in a BD Difco Nutrient broth supplemented with 5 g/L yeast extract (NYB).

### Antibiotic susceptibility measurement by culturing

Thawed cells from 20% glycerol stocks of the test strain was diluted in NYB, cultured 18 hours at 30°C and diluted in a fresh medium to an optical density (OD)_660nm_ of 0.005. An antibiotic chloramphenicol (Fujifilm Wako Pure Chemical Corporation, Osaka, Japan) or ofloxacin (Fujifilm Wako Pure Chemical Corporation) was added to the bacterial suspension to achieve a final concentration of 0.25–32 µg/mL, and the suspension was incubated at 30°C in standing culture for 24 hours. The OD_660nm_ was measured using a plate reader. After culture, the minimum concentration at which the OD_660nm_ did not exceed 0.02 was taken as the minimum inhibitory concentration [7].

### Preparation of DNA samples for Nanopore sequencer performance evaluation

The test strain was cultured at 30°C 18 hours, and the genomic DNA was extracted using the ZymoBIOMICS DNA Miniprep Kit (Zymo Research, California, USA) according to manufacturer’s protocol. Polymerase chain reaction (PCR) was performed using primers that bind to both ends of the 16S rRNA gene (27F primer [21]: 5’- AGAGTTTGATCCTGGCTCAG-3’, 1492R primer [22]: 5’-GGTTACCTTGTTACGACTT-3’) and KAPA HiFi HotStart ReadyMix (Kapa Biosystems, Wilmington, MA). The PCR thermal profile consisted of an initial denaturation of 4 min at 95°C, followed by 30 cycles of 20 s at 98°C, 15 s at 60°C, 30 s at 72°C, and a final step of 2 min at 72°C. The amplification product was purified using the FastGene Gel/PCR Extraction Kit (NIPPON Genetics, Tokyo, Japan) according to manufacturer’s protocol and the concentration was determined using a Qubit ® Fluorometer (Thermo Fisher Scientific, Waltham, MA). Nanopore sequencing samples were prepared to check the taxonomy identification performance (Table 1) using the 16S rRNA gene amplification product of each strain separately, and to check the accuracy of abundance ratio determination (Table 2) using a mixture of the 16S rRNA gene amplification products in a given ratio. The concept of measuring the absolute amount of each 16S rRNA was also confirmed using 16S rRNA amplicons prepared from eight different test bacteria. All eight test amplicons were mixed at equal concentrations to achieve the desired amount of concentration shown in A to D (Sample A = 1.11 µg/mL, Sample B = 0.22 µg/mL, Sample C = 0.044 µg/mL, Sample D = 0.0089 µg/mL, Table 3). The 16S rRNA amplicon of *R*. *solanacearum* in a final concentration of 1.11 µg/mL was added to each sample as an internal standard.

**Table 1:** Proportion of accurately identified reads of test strains analyzed using the Nanopore sequencing.

Test strain	Family level	Genus level	Species level
*Kocuria rhizophila*	98.1	97.8	92.3
*Escherichia coli*	98.7	77.0	0.2
*Enterococcus hirae*	99.3	99.3	96.4
*Bacillus subtilis*	99.1	99.0	86.5
*Enterococcus faecalis*	99.2	99.1	97.5
*Streptococcus mutans*	99.6	99.4	99.0
*Ralstonia solanacearum*	99.2	98.8	46.4
*Listeria innocua*	99.0	98.9	18.3
*Staphylococcus epidermidis*	98.8	98.7	40.7

The 16S rRNA sequence reads of nine bacteria obtained using Nanopore sequencer were searched for homology with rRNA database and classified into the species, genus, and family level.

**Table 2:** Detection of the relative abundances in samples of mixed bacteria with different abundance ratios.

Strain	Calculated (%)	Detected (%)
*Ralstonia*	50.1	34.8
*Kocuria*	25.0	42.4
*Enterobacteriaceae*	12.5	8.2
*Bacillus*	6.3	6.7
*Staphylococcus*	3.1	2.3
*Listeria*	1.6	1.2
*Enterococcus faecalis*	0.78	0.85
*Enterococcus hirae*	0.39	0.36
*Streptococcus*	0.20	0.17

*E*. *coli* was classified as *Enterobacteriaceae* family.

**Table 3:** The absolute amount quantification of the gene using an internal standard.

Strain	Sample A (µg/mL)	Sample B (µg/mL)	Sample C (µg/mL)	Sample D (µg/mL)
*Kocuria*	0.85	0.17	0.039	0.0071
*Enterobacteriaceae*	0.84	0.16	0.034	0.0060
*Enterococcus hirae*	0.98	0.21	0.037	0.0088
*Bacillus*	0.86	0.17	0.036	0.0064
*Enterococcus faecalis*	0.87	0.16	0.034	0.0057
*Streptococcus*	0.79	0.16	0.038	0.0069
*Listeria*	0.89	0.17	0.037	0.0073
*Staphylococcus*	0.95	0.17	0.035	0.0066
Theoretical value	1.11	0.22	0.044	0.0089

The absolute gene concentrations of the various test strains were calculated from the ratio of the number of rRNA gene reads in the Ralstonia internal standard (1.1 μg/mL) to the number of rRNA gene reads in the test strain. *E*. *coli* was classified as *Enterobacteriaceae* family.

### Preparation of mix culture samples for antimicrobial susceptibility test by sequencing

The test strain that cultured 18 hours at 30°C was centrifuged, the resulting supernatant was removed, cells were resuspended in fresh medium and the suspension of all strains were mixed to contain equal amounts of the rRNA gene at an OD_660nm_ of 0.2. The number of rRNA genes was calculated by multiplying the number of colony-forming units (CFUs) by the copy number of the 16S rRNA gene present in each strain. CFUs were counted as below. The test strains were incubated for 18 hours at 30°C in NYB medium, each strain was collected by centrifugation (2 min, 13,000 g), washed with phosphate-buffered saline, serially diluted in phosphate-buffered saline, and 20 µL was spotted 3 times onto agar plates prepared with ATCC recommended medium of each strain. Plates were incubated for 24 hours at 30°C and CFUs were measured (20–100 colonies were counted for each strain). The antibiotic chloramphenicol or ofloxacin was added to the mixed culture, and the strain was incubated in standing culture at 30°C for 6 hours. After incubation, a known bacterium was added as the internal standard for absolute quantitation. The internal standard consisted of 25 µL of a suspension of *Lactobacillus sakei* at an OD_660nm_ of 2.0. The use of a taxonomically distant strain as the internal standard facilitates the identification of the reads from the internal standards. We selected *L*. *sakei* because it differs at least at the family level from each of the test strains. The DNA was then extracted using the ZymoBIOMICS DNA Miniprep Kit to prepare a library following the manufacturer’s protocol.

### Library preparation and data analysis for Nanopore sequencing

The 16S rRNA amplicon Library preparation was performed using the 16S Barcoding Kit 1–24 (Oxford Nanopore Technologies, Oxford, UK) according to the manufacturer’s protocol. The PCRs were conducted using a primer contained in the kit and LongAmp Taq 2X Master Mix. After purifying the PCR product with Agencourt AMPure XP beads (Beckman Coulter, California, USA), the amount of eluted DNA was determined using Qubit® Fluorometer. PCR amplicon (100 ng of DNA in 10 µL buffer) was added to 1 µL of rapid adapter (Oxford Nanopore Technologies) and incubated at room temperature according to the manufacturer’s protocol of Rapid Sequencing Kit (Oxford Nanopore Technologies). Sequencing solution was prepared by mixing amplicon library (11 µL), sequencing buffer (34 µL), loading beads (25.5 µL) and water (4.5 µL), and loaded on MinION R9.4.1 SpotON Flow Cells. The sequencer was controlled using the MinKNOW software v3.6.5. For each sample, more than 120,000 reads having a Q-score exceeding 7 were acquired and used for the analysis. Fast5 files were base-called, de-multiplexed and trimmed barcode and adapter sequences using guppy v3.2.10 (Oxford Nanopore Technologies). The base-called sequences (FASTQ files) were mapped to the database of bacterial 16S rRNA sequences from NCBI RefSeq Targeted Loci Project using Minimap2 [23]. Genus names, taxonomy identifiers, and read counts were listed using our original program. The absolute abundance of each test strain was determined by comparing read count of test strain with that of internal standard of known concentration. The fold change of absolute bacterial abundances after exposure to antibiotics comparing with before exposure was showed as bacterial reduction due to antibiotics.

## Results

### Performance evaluation of Nanopore sequencing in taxonomy identification

To evaluate the performance of the taxonomy identification, the rRNA genes from clones of known test strains were amplified, purified and analyzed with the Nanopore sequencer. Reads from nine test strains were classified into the family, genus, and species levels and the proportion of reads with the correct classifications were calculated (Table 1). At the family level, all strains showed high accuracy of 98% to 99%. At the genus level, all strains except for *Escherichia* also revealed high accuracies of 98% to 99%, which is nearly the accuracy at the family level. For *Escherichia*, 18.4% of reads were classified as the *Shigella* genus in the same *Enterobacteriaceae* family. The accuracy of identification at the species level varied depending on the strain, and *E*. *hirae* and *E*. *faecalis* could be identified at high accuracy of 96.4% and 97.5%, respectively. In the following analyses, *E*. *coli* were classified at the family level, *E*. *hirae* and *E*. *faecalis* at the species level, and other bacteria at the genus level. Next, to determine the accuracy of the abundance ratio measurement, a mixture containing 16S rRNA genes from nine strains in a known ratio were sequenced. When the abundance of each strain was 0.2–50% (Table 2), the rRNA genes of all strains were detected. In particular, it was found that the presence ratio of even low concentration sequences was accurately detected, although some errors in abundance ratio were noted. Even at an abundance of about 1%, *E*. *hirae* and *E*. *faecalis* were distinctly identified.

Since current methods cannot determine the change in the absolute amount of bacteria in the culture, although they can determine the presence ratio, we attempted to quantify the absolute amount of the gene using an internal standard. Equal amounts of the 16S rRNA genes from eight strains were mixed to prepare mixtures at the total concentration of 0.0088–1.1 µg/mL, and the mixtures were supplemented with 1.1 µg/mL of the *Ralstonia* rRNA gene as the internal standard and analyzed using the Nanopore sequencer (Table 3). All genes of the test strains, from Sample A (at nearly the same concentration as in the internal standard) to Sample D (containing the genes of the test strains at 1/125 compared with the internal standard), were detected with not more than 50% deviation from the theoretical values.

### Physiological properties of the test strains

Since the Nanopore sequencer could be used for bacterial identification at the species level and determination of absolute abundance, we considered it would be applicable to the evaluation of antibiotic sensitivity of mixed bacteria. Prior to mixing, the physiological properties of each test strain were examined. According to ATCC recommendations, various strains require different media for growth including Luria–Bertani medium, MRS broth, tryptic soy broth, nutrient broth (NB), and brain heart infusion broth (BHI). When nine test strains were cultured separately in each medium, some test strains proliferated explosively while others slowly, indicating that there were several orders of growth rate differences. Because it was likely that, when mixed, fast-proliferating strains would consume all nutrients and others die without the use of antibiotics, we searched for media that would allow all strains to proliferate at similar rates. When cultured in a NB supplemented with yeast extract (NYB) for 6 hours, each test strain increased by 4–16 fold (S1 Fig). Since the proliferation rate difference between strains was only up to 4-fold, this medium was selected for use in the mixed culture.

To determine the concentration of antibiotics to use in the mixed culture, each test strain was cultured separately and sensitivities to the antibiotics ofloxacin and chloramphenicol were measured (Table 4). The test strains were susceptible against ofloxacin at concentrations of 0.25–8 µg/mL and chloramphenicol at concentrations of 2–8 µg/mL.

**Table 4:** Minimum inhibitory concentration (MIC) of ofloxacin and chloramphenicol against test strain cultured in NYB.

Strain	Ofloxacin (µg/mL)	Chloramphenicol (µg/mL)
*Kocuria rhizophila*	8	2
*Escherichia coli*	1	4
*Enterococcus hirae*	2	4
*Bacillus subtilis*	0.5	2
*Enterococcus faecalis*	16	4
*Streptococcus mutans*	8	2
*Ralstonia solanacearum*	<0.25	8
*Listeria innocua*	8	8
*Staphylococcus epidermidis*	1	4

### Measuring antibiotic sensitivity in mixed culture

**Fig 2:**
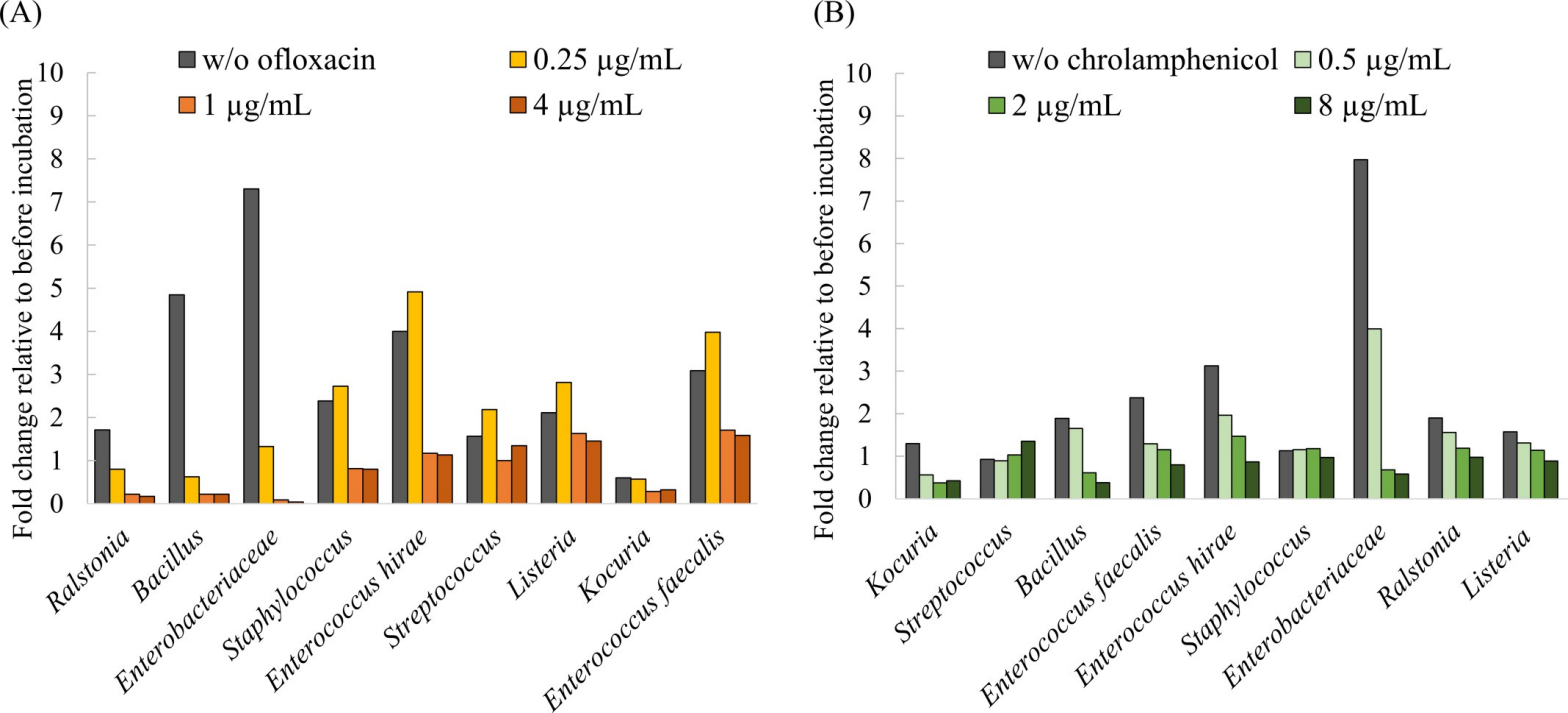
Antimicrobial susceptibility test using Nanopore sequencing. The test strains mixture was exposed to (A) ofloxacin or (B) chrolamphenicol, and incubated 6 hours. The absolute abundance of each test strain was calculated by comparing sequencing reads of test strain and internal standard strain. The abundances fold change of after incubation relative to before incubation were shown in Y axis. The strains are arranged in descending order of antibiotic sensitivity from left to right (see Table 4). *E*. *coli* was classified as *Enterobacteriaceae* family.

The proliferation of mixed-culture bacteria was measured using the Nanopore sequencer. When mixed to include equivalent amounts of the 16S rRNA gene and cultured, all strains were found to have proliferated 1.07- to 19.7-fold compared with initial counts (S2 Fig). In the presence of ofloxacin (Fig 2A), the proliferation of *Ralstonia*, *Bacillus* and *Enterobacteriaceae*, which are highly sensitive to ofloxacin (Table 4), decreased remarkably at low concentration (0.25 µg/mL) compared with the control, whereas the other strains showed slightly increased growth. Increasing the ofloxacin concentration to 1 and 4 µg/mL suppressed proliferation of all test strains. In addition, when *Ralstonia*, *Bacillus* and *Enterobacteriaceae* were exposed to high concentrations of ofloxacin, the numbers of their reads were much smaller than the initial numbers (4% of the initial reads). When chloramphenicol was added (Fig 2B), on the other hand, the growth of some strains was suppressed by about 50% even at low concentrations, and higher concentrations showed stronger suppressions. In contrast to ofloxacin, no strains decreased dramatically. All the DNA sequences generated in the present study are available from the DDBJ Sequence Read Archive (DRA) database (DRA012954).

## Discussion

### The advantage and disadvantage of Nanopore sequencing compared to NGS in taxonomy identification

The performance of Nanopore sequencing in taxonomy identification was evaluated in this study. The advantage of the Nanopore sequencer over NGS is the ability to identify bacterial taxonomy at the genus or species level. Conventional 16S rRNA amplicon sequencing targets a short fragment of the 16S rRNA gene, commonly limited to the V3–V4 region (460 bp) alone, which is an obstacle to achieving the depth of identification. Some studies suggest full length of the 16S rRNA gene is needed for bacterial identification at the genus and species levels [24,25]. Analysis of the full length of 16S rRNA by Nanopore sequencing allowed us to assign taxonomy down to species level. In this study, *E*. *faecalis* and *E*. *hirae* were identified at the species level at an accuracy of up to 98%. All strains except for *Escherichia*, which are difficult to distinguish because of their high sequence homology with *Shigella* [26], could be identified at the genus level with high accuracies of 98–99%. These results demonstrated that the Nanopore sequencing allowed for convenient taxonomy identification of bacteria from a mixture containing closely related species even at the species or genus level.

The disadvantages of the Nanopore sequencer compared to NGS is higher misclassification rates. Although previous studies suggested that the Nanopore sequencing could identify some bacteria of the genus *Streptococcus* or *Staphylococcus* at the species level [14–16], it was unknown how phylogenetically distant species each test species might be wrongly classified as, because these studies classified bacteria in a predefined set of species. Our analysis of the 16S rRNA gene from one strain revealed that 0.2% of the reads were classified into wrong phyla, probably because of higher error rates of the third-generation sequencer than the Illumina platform. Therefore, very rare populations (less than 0.2% of all leads) might be strongly affected by misidentification and Nanopore sequencing is not suitable for applications where such populations are important. To improve the read accuracy, such as base-caller development, R10 flow cells or obtaining longer read length [15,27], are required for accurate quantification of very rare populations.

### Quickness and convenience of this approach

The 16S barcoding kit includes 24 barcode sequences, enabling an examination of 24 conditions × 9 bacterial species (216 reactions) in a single experiment. In this study, we acquired 120,000 reads per sample by 18 hours sequencing, whereas Mitsuhashi et al. [14] reported bacterial detection by 5-min sequencing using the MinION platform. The analysis was completed in several minutes per sample by using minimap2 in our computing environment (an AWS EC2 instance with 36 vCPUs). Therefore, with this technique using the Nanopore sequencer, we can obtain more extensive data in shorter time periods than the conventional approaches of the disk assay method and the microdilution method.

### Determination of absolute abundance of test strains in mixed culture by Nanopore sequencing

As we were able to quantify purified rRNA gene amplification products at the species level by using Nanopore sequencing and our analytical method, we then tested whether the absolute amounts of genes could be quantified in a medium containing multiple bacterial strains. This technique requires three conditions. First, the difference in DNA extraction efficiency among different bacteria must be minimized. This condition could be satisfied using the ZymoBIOMICS DNA Miniprep Kit, which has been shown to be an effective extraction method for 16S rRNA amplicon analysis referred in several papers [28,29].

Second, the absolute amounts must be quantified, which could be achieved with an internal standard of bacterial cells added at known concentrations. Such a method for microbial community analysis had been used with NGS [19], but not with Nanopore sequencer. Although there were concerns about decreased quantitation performance due to identification errors and PCR biases stemming from differences in the sequencing method and the method of library preparation, quantitation of purified rRNA genes showed that samples with a 100-fold difference in concentrations could be quantitated correctly (Table 3). This finding showed that using an internal standard for bacterial quantitation was applicable to 16S rRNA gene analysis using the Nanopore sequencer.

Third, it is necessary to select a medium that allows concurrent growth of multiple bacterial species because the appropriate nutrient medium for growth differs among bacterial species. To find an appropriate medium, we cultured all test strains using Luria–Bertani medium, MRS broth, tryptic soy broth, NB, and brain heart infusion broth recommended for the respective strains, and found that NB produced smallest differences in proliferation rate among the strains. Although *S*. *mutans* did not proliferate in NB at all, the proliferation rates in other media suggested that this strain could proliferate in media containing yeast extract. Therefore, we prepared NB supplemented with yeast extract (NYB), and confirmed that all of the test strains grew equally in this medium. We considered that bactericidal action and bacteriostatic action could be distinguished by using the medium.

### Applicability of Nanopore based antibiotic susceptibility testing

We showed that the antibiotic sensitivities of nine bacterial strains in mixed culture could be measured using a Nanopore sequencer. When ofloxacin was added at a concentration of 0.25 µg/mL, only the level of highly sensitive bacteria, i.e., *Ralstonia*, *Bacillus*, and *Enterobacteriaceae*, decreased markedly, whereas that of the other strains increased slightly. This result suggested that the antibiotic had little impact on the six surviving strains, and that their proliferation rates increased as a result of the availability of nutrients that would have been consumed by the three strains killed by the antibiotic. When the ofloxacin concentration was increased, none of the strains proliferated, and three strains of *Ralstonia*, *Bacillus*, and *Enterobacteriaceae*, decreased to 4% of the initial amount. This result, combined with the fact that ofloxacin is an antibiotic that kills bacteria by inhibiting their DNA gyrase [30], suggested that the bacteria exposed to high-concentration ofloxacin had their cell membranes damaged by lysis or sterilization, resulting in elution of the genomic DNA into the medium and washed away in the library preparation. Although chloramphenicol also caused concentration-dependent reductions in the proliferation rate, the bacterial count became no less than 0.4 of the initial count even in the most sensitive strain *Kocuria*. Chloramphenicol is known as a bacteriostatic agent that binds to ribosomes to stop protein synthesis [31], and the above finding suggests that bacterial killing did not occur as with ofloxacin. The decrease in bacterial count from the initial count despite the bacteriostatic effect is attributable to stress due to competition with other strains in the bacterial mixture. When bacterial proliferation was measured in antibiotic-free mixed culture (S2 Fig), the count of slowly proliferating *Kocuria* decreased from the initial count. This bacterium might have died gradually under stress from other bacteria when its proliferation was inhibited by chloramphenicol. For the test strains in this study, the bacterial count reduction due to antibiotics differed 10 fold between the bactericidal antibiotic (ofloxacin) and the bacteriostatic antibiotic (chloramphenicol). However, to apply the present technique to other bacteria or novel antibiotic candidates, it is necessary to clarify the boundary statistically between bactericidal and bacteriostatic antimicrobials using known antibiotics. Moreover, caution should be exercised to prevent the occurrence of bacterial death by bacteriocin [32] or the type VI secretion system [33] due to strain interactions in the mixture.

In conclusion, this study demonstrated the utility of Nanopore sequencing of 16S rRNA amplicons as an approach to evaluate the antimicrobial susceptibility by analyzing the absolute abundance of test species. Although conventional method by 16S rRNA amplicon sequence can only analyze the relative amounts of bacteria, the absolute abundances of all species in a bacterial mixture can be measured simultaneously just by spiking the sample with an internal standard strain. Our method is quicker than colony counting, and is applicable to an antimicrobial susceptibility testing that can distinguish between bactericidal and bacteriostatic effects. Further improvements in the mixed culture conditions to ensure survival of the test species will allow for application of the method to various bacteria, such as biofilm bacteria and gut microbiota.

## Supporting information

S1 Fig Proliferation speeds of test strains in NYB. (TIF)

S2 Fig Increases in cell counts of various strains in mixed culture in the absence of antibiotics, measured using Nanopore sequencing. The test strains mixture was incubated 6 hours. The absolute abundance of each test strain was calculated by comparing sequencing reads of test strain and internal standard strain. The abundances fold change of after incubation relative to before incubation were shown in Y axis.
(TIF)
