# Supplementary figures and images for: Development of an efficient antimicrobial susceptibility testing method with species identification by Nanopore sequencing of 16S rRNA amplicons
Source: PLoS One. 2022 Feb 3;17(2):e0262912. doi: 10.1371/journal.pone.0262912 (PMC8812843; doi:10.1371/journal.pone.0262912)

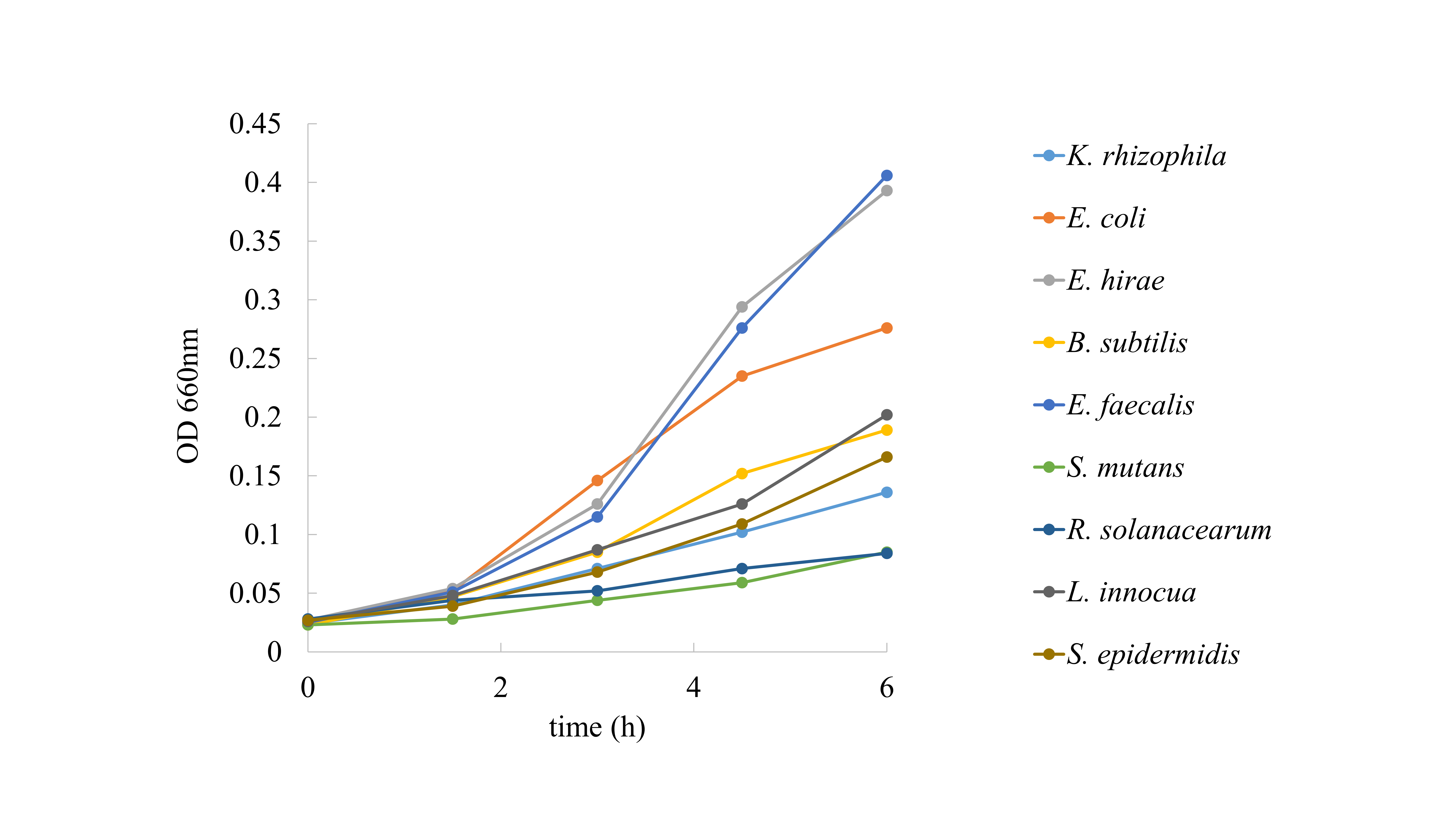

Supplement: S1 Fig — (TIF) [file pone.0262912.s001.tif]

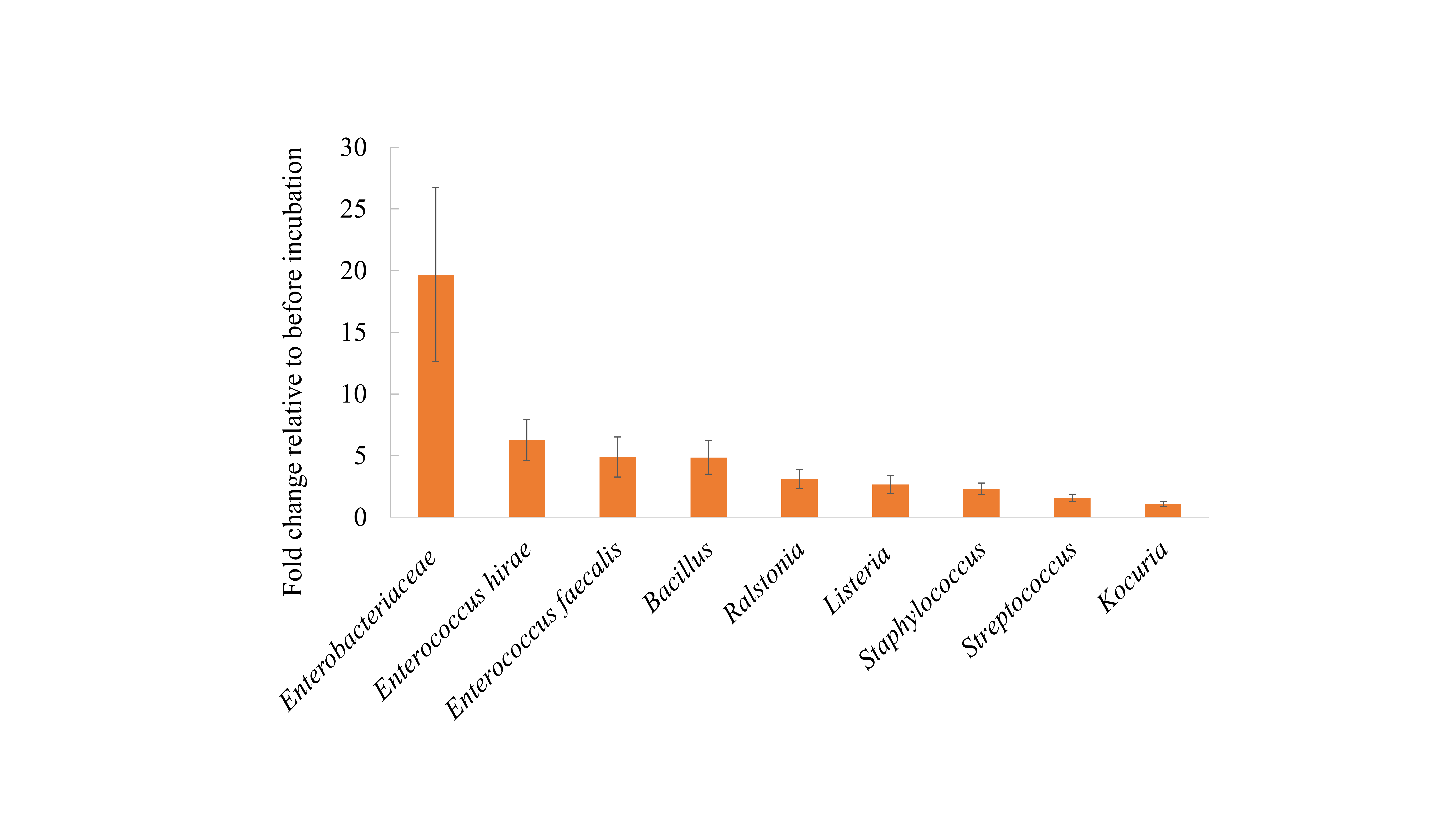

Supplement: S2 Fig — The test strains mixture was incubated 6 hours. The absolute abundance of each test strain was calculated by comparing sequencing reads of test strain and internal standard strain. The abundances fold change of after incubation relative to before incubation were shown in Y axis. (TIF) [file pone.0262912.s002.tif]
